# Supplementary material for: Regulations of Citrus Pectin Oligosaccharide on Cholesterol Metabolism: Insights from Integrative Analysis of Gut Microbiota and Metabolites
Source: Nutrients. 2024 Jun 24;16(13):2002. doi: 10.3390/nu16132002 (PMC11243408; doi:10.3390/nu16132002)
Supplement: Supplementary file 1 [file nutrients-16-02002-s001.zip › nutrients-2950894-supplementary.pdf]

Supplementary Materials for

Regulations of Citrus Pectin Oligosaccharide on Cholesterol Metabolism: Insights from Integrative Analysis of Gut Microbiota and Metabolites

Haijuan Hu [1,2], Peipei Zhang [1,2], Fengxia Liu [1,2] and Siyi Pan [1,2,*]

[1] College of Food Science and Technology, Huazhong Agricultural University, Wuhan 430070, China;
huhaijuan@163.com (H.H.); zhangpeipei1217@webmail.hzau.edu.cn (P.Z.);
liufxia@mail.hzau.edu.cn (F.L.)

[2] Key Laboratory of Environment Correlative Dietology, Ministry of Education, Huazhong Agricultural
University, Wuhan 430070, China

* Correspondence: siyipan401@163.com; Tel.: +86-13554029828

SUPPLEMENTAL EXPERIMENTAL PROCEDURES

In vitro fermentation. In vitro fermentation was performed according to our previous report [28]. Briefly, human pooled feces were cultured with or without 5% (w/ v) POSH1 samples at 37.0 °C for 24.0 h in an anaerobic chamber. At 24.0 h, supernatants and sediments of bacterial sludge were obtained from centrifugation. Supernatants were filter sterilized using a Millipore Express filter unit (0.22 μm pore diameter) and defined as N24 (background medium without POSH1; P24_1, P24_2, P24_3, P24_4, P24_5, and P24_6) or P24 (background medium with POS_{H1} substrate; (N24_1, N24_2, N24_3, N24_4, N24_5, and N24_6)), respectively. The sediment of bacterial sludge obtained from the control group was denoted as group N, which consisted of 6 samples (N1, N2, N3, N4, N5, and N6). Sediments of bacterial sludge obtained from the POS_{H1} group was denoted as group P, which consisted of 6 samples (P1, P2, P3, P4, P5, and P6). Sediments of bacterial sludge were stored at -80°C for subsequent Illumina Miseq sequencing. The supernatants were filtered with 0.22 μm membrane and stored at -80°C for subsequent non-targeted metabolomics and SCFAs detection to evaluate the changes of intestinal microbial fermentation products.

DNA extraction, PCR amplification and Illumina MiSeq sequencing. The DNA extract was checked on 1% agarose gel, and DNA concentration and purity were determined with NanoDrop 2000 UV-vis spectrophotometer (Thermo Scientific, Wilmington, USA). The hypervariable region V3-V4 of the bacterial 16S rRNA gene were amplified with primer pairs 338F (5'-ACTCCTACGGGAGGCAGCAG-3') and 806R (5'-GGACTACHVGGGTWTCTAAT-3') by an ABI GeneAmp® 9700 PCR thermocycler (ABI, CA, USA). The PCR amplification of 16S rRNA gene was

performed as follows: initial denaturation at 95 ℃ for 3 min, followed by 27 cycles of denaturing at 95 ℃ for 30 s, annealing at 55 ℃ for 30 s and extension at 72 ℃for 45 s, and single extension at 72 ℃ for 10 min, and end at 4 ℃. The PCR mixtures contain 5×TransStart FastPfu buffer 4 μL, 2.5 mM dNTPs 2 μL, forward primer (5 μM) 0.8 μL, reverse primer (5 μM) 0.8 μL, TransStart FastPfu DNA Polymerase 0.4 μL, template DNA 10 ng, and finally ddH2O up to 20 μL. PCR reactions were performed in triplicate. The PCR product was extracted from 2% agarose gel and purified using the AxyPrep DNA Gel Extraction Kit (Axygen Biosciences, Union City, CA, USA) according to manufacturer's instructions and quantified using Quantus™ Fluorometer (Promega, USA)

Purified amplicons were pooled in equimolar and paired-end sequenced on an Illumina MiSeq PE300 platform/NovaSeq PE250 platform (Illumina, San Diego,USA) according to the standard protocols by MajorbioBio-Pharm Technology Co. Ltd. (Shanghai, China).

Processing of sequencing data. The raw 16S rRNA gene sequencing reads were demultiplexed, quality-filtered by fastp version 0.20.0 [29], and merged by FLASH version 1.2.7 with the following criteria [30]: (i) the 300 bp reads were truncated at any site receiving an average quality score of <20 over a 50 bp sliding window, and the truncated reads shorter than 50 bp were discarded, reads containing ambiguous characters were also discarded; (ii) only overlapping sequences longer than 10 bp were assembled according to their overlapped sequence. The maximum mismatch ratio of overlap region is 0.2. Reads that could not be assembled were discarded; (iii)

Samples were distinguished according to the barcode and primers, and the sequence direction was adjusted, exact barcode matching, 2 nucleotide mismatches in primer matching.

Operational taxonomic units (OTUs) with 97% similarity cutoff were clustered using UPARSE version 7.1[64, 65], and chimeric sequences were identified and removed. The taxonomy of each OTU representative sequence was analyzed by RDP Classifier version 2.2 against the 16S rRNA database (eg. Silva v138) using confidence threshold of 0.7 [66]. The Shannon index, Simpson index, Chao index, and Ace index were used to describe the α-diversity index.

Metabolite extraction and UPLC-MS/MS analysis. 200 uL liquid sample were accurately weighed, and the metabolites extracted using a 400 μL methanol: water (4:1, v/v) solution. The mixture was allowed to settle at -20℃ and treated by High throughput tissue crusher Wonbio-96c (Shanghai wanbo biotechnology co., LTD) at 50 Hz for 6 min, then followed by vortex for 30s and ultrasound at 40 kHz for 30 min at 5℃. The samples were placed at -20℃ for 30 min to precipitate proteins. After centrifugation at 13000g at 4℃ for 15min, the supernatants were carefully transferred to sample vials for LC-MS/MS analysis.

Chromatographic separation of the metabolites was performed on a ExionLCTMAD system (AB Sciex, USA) equipped with an ACQUITY UPLC HSS T3 column (100 mm × 2.1 mm i.d., 1.8 μm; Waters, Milford, USA). The mobile phases consisted of 0.1% formic acid in water with formic acid (0.1%) (solvent A) and 0.1% formic acid in acetonitrile: isopropanol (1:1, v/v) (solvent B). The solvent

gradient changed according to the following conditions: from 0 to 3 min, 95% (A): 5% (B) to 80% (A): 20% (B); from 3 to 9 min, 80% (A): 20% (B) to 5% (A): 95% (B); from 9 to 13 min, 5% (A): 95% (B) to 5% (A): 95% (B) ; from 13 to 13.1 min, 5% (A): 95% (B) to 95% (A): 5% (B), from 13.1 to 16 min, 95% (A): 5% (B) to 95% (A): 5% (B) for equilibrating the systems. The sample injection volume was 20 uL and the flow rate was set to 0.4 mL/min. The column temperature was maintained at 40 ℃. During the period of analysis, all these samples were stored at 4 ℃.

The UPLC system was coupled to a quadrupole-time-of-flight mass spectrometer (Triple TOFTM5600+, AB Sciex, USA) equipped with an electrospray ionization (ESI) source operating in positive mode and negative mode. The optimal conditions were set as followed: source temperature, 500 ℃; curtain gas (CUR), 30 psi; both Ion Source GS1 and GS2, 50 psi; ion-spray voltage floating (ISVF), -4000V in negative mode and 5000V in positive mode, respectively; declustering potential, 80V; a collision energy (CE), 20-60V rolling for MS/MS. Data acquisition was performed with the Data Dependent Acquisition (DDA) mode. The detection was carried out over a mass range of 50-1000 m/z.

Data preprocessing and annotation. After UPLC-TOF/MS analyses, the raw data were imported into the Progenesis QI 2.3 (Nonlinear Dynamics, Waters, USA) for peak detection and alignment. The preprocessing results generated a data matrix that consisted of the retention time (RT), mass-to-charge ratio (m/z) values, and peak intensity. Metabolic features detected at least 80 % in any set of samples were retained. After filtering, minimum metabolite values were imputed for specific

samples in which the metabolite levels fell below the lower limit of quantitation and each Metabolic features were normalized by sum. The internal standard was used for data QC (reproducibility), Metabolic features which the relative standard deviation (RSD) of QC>30% were discarded. Following normalization procedures and imputation, statistical analysis was performed on log transformed data to identify significant differences in metabolite levels between comparable groups. Mass spectra of these metabolic features were identified by using the accurate mass, MS/MS fragments spectra and isotope ratio difference with searching in reliable biochemical databases as Human metabolome database (HMDB) (http://www.hmdb.ca/) and Metlin database (https://metlin.scripps.edu/).

Multivariate statistical analysis.

A multivariate statistical analysis was performed using ropls (Version1.6.2, http://bioconductor.org/packages/release/bioc/html/ropls.html) R package from Bioconductor on Majorbio Cloud Platform (https://cloud.majorbio.com). Principle component analysis (PCA) using an unsupervised method was applied to obtain an overview of the metabolic data, general clustering, trends, or outliers were visualized. All of the metabolite variables were scaled to unit-variances prior to conducting the PCA. Partial least squares discriminate analysis (PLS-DA) was used for statistical analysis to determine global metabolic changes between comparable groups. All of the metabolite variables were scaled to pareto Scaling prior to conducting the PLS-DA. The model validity was evaluated from model parameters R^2 and Q^2, which provide information for the interpretability and predictability, respectively, of the

model and aviod the risk of over-fitting. Variable importance in the projection (VIP) were calculated in PLS-DA model. p values were estimated with paired Student's t-test on Single dimensional statistical analysis.

Differential metabolites analysis. Statistically significant among groups were selected with VIP value more than 1 and p value less than 0.05. Differential metabolites among two groups were summarized, and mapped into their biochemical pathways through metabolic enrichment and pathway analysis based on database search (KEGG, http://www. genome.jp/kegg/). These metabolites can be classified according to the pathways they involved or the functions they performed. Enrichment analysis was usually to analyze a group of metabolites in a function node whether appears or not. The principle was that the annotation analysis of a single metabolite develops into an annotation analysis of a group of metabolites. scipy. stats (https://docs.scipy.org/doc/scipy/) was exploited to identify statistically significantly enriched pathway using Fisher's exact test.

Determination of the concentration of SCFAs in intestinal flora fermentation products. Take 0.5 g of the supernatant sample in a 10 mL centrifuge tube, add 2 mL of water (10% phosphoric acid aqueous solution), stir to mix the sample, add 2 mL of ether for extraction for 5 min, and then centrifuge at 4000 r/min for 15 min. min; after centrifugation, take the ether phase, then add 1 mL of ether to extract twice according to the same procedure, combine the three extracts, constant volume, and inject for analysis.

Prepare different concentrations (0 mg/L, 10 mg/L, 20 mg/L, 30 mg/L, 40 mg/L,

50 mg/L, 60 mg/L, 70 mg/L and 80 mg/L mg/L) SCFAs mixed standard solution (acetic acid, propionic acid, butyric acid, isobutyric acid, valeric acid, isovaleric acid). The SCFAs mixed standard was processed in the same way as the sample, and the samples were injected sequentially to draw a standard curve (R^2=0.99) for each SCFAs with a concentration of 0-80 mg/L, and the final concentration in the supernatant was converted to mg/kg.

Agilent TG WAX capillary gas chromatography column (30 m × 0.25 mm × 0.25 μm); split ratio 75:1, injection volume 1 μL; inlet temperature 240 °C, ion source temperature 200 °C, transfer line temperature 250 °C; the carrier gas is helium, and the flow rate is 1 mL/min; the heating program of the chromatographic column is as follows: the initial 100 °C is maintained for 5 min, the temperature is raised to 150 °C at 5 °C/min, and then the temperature is raised at 30 °C/min to 240 °C for 30 min.
